# Evaluation of inter‐fractional anatomical variations due to compression belt placement reproducibility using MR‐guided radiotherapy treatment fraction images

**DOI:** 10.1002/acm2.70783

**Published:** 2026-09-03

**Authors:** Olivia Shay, David Solis, Morgan Aire, Christopher Schneider, Krystal Kirby

**Affiliations:** ^1^ Department of Physics and Astronomy Louisiana State University Baton Rouge Louisiana USA; ^2^ Department of Radiation Oncology Mary Bird Perkins Cancer Center Baton Rouge Louisiana USA

**Keywords:** Adaptive Therapy, Compression Belt, Motion Management

## Abstract

**Background:**

Abdominal compression (AC) using a belt‐based system is widely employed in radiation therapy to limit respiration‐induced target motion for abdominal and hepatic malignancies. While AC has demonstrated efficacy in reducing intrafraction motion amplitude, the interfractional anatomical consequences of daily compression belt (CB) placement variability remain poorly characterized. A clearer understanding of this variability is necessary to determine whether online adaptive radiotherapy (oART) is required to maintain dosimetric robustness throughout the treatment course.

**Purpose:**

This study aims to quantify interfractional anatomical variability associated with CB use in a clinical MR‐guided radiotherapy cohort and to compare these findings against a matched cohort of non‐compressed abdominal patients to determine the clinical significance of CB‐induced setup variability.

**Methods:**

Daily treatment MR images were retrospectively collected from 17 CB patients and 17 matched non‐CB controls treated on an MR‐Linac. External contours were delineated at the center of the planning target volume (PTV) and at the region of maximum compression for CB patients. Interfractional variability was assessed using cross‐sectional area (CSA), Dice Similarity Coefficient (DSC), Mean Distance to Agreement (MDA), and Hausdorff Distance (HD). Interfractional organ displacement of the liver and kidneys was quantified via rigid registration. Linear mixed‐effects models (LMEMs) evaluated the association between patient demographics—including age, BMI, sex, subcutaneous adipose tissue (SAT), visceral adipose tissue (VAT)—and interfractional variability metrics.

**Results:**

Interfractional DSC over the PTV region was significantly lower in the CB cohort compared with non‐CB controls (*p* = 0.01 and *p* = 0.03 for comparisons to fraction one and sequential fraction comparisons, respectively), and MDA was significantly elevated in the CB cohort (*p* < 0.001). No significant difference in CSA variability was observed between cohorts. In the maximum compression region, median HD was 1.6 cm relative to fraction one, with no significant correlation between compression‐region and PTV‐region CSA variability (*p* = 0.99). Older age, male sex, and higher BMI were associated with improved belt‐contact shape reproducibility (all *p* < 0.05), though these demographic effects did not translate to PTV‐region stability. LMEM analysis revealed that fixed demographic effects explained only a small fraction of PTV variability (R^2^_m_ = 0.06–0.29), with the remainder attributable to patient‐specific random effects. Interfractional organ displacement ranged from 5.91 to 9.15 mm across organs and cohorts, with no statistically significant difference between groups.

**Conclusions:**

CB use introduces measurable but modest increases in interfractional anatomical variability at the PTV level compared with standard abdominal setups. Compression‐site variability does not propagate directly to the treatment target, and patient demographics alone are insufficient to predict CB‐induced setup variability. Daily MR‐based image guidance and selective adaptive replanning remain the appropriate management strategy for patients treated with abdominal compression.

## 1 INTRODUCTION

In radiation therapy, geometric margins are applied to the clinical target volume to ensure that the prescribed dose adequately encompasses the tumor throughout the entire treatment course.^1^, ^2^ These margins compensate for setup and machine uncertainties, as well as physiological motion, particularly respiration‐induced displacement. However, enlarging the treatment volume by adding these margins increases the irradiated volume of healthy tissue, which may elevate toxicity risk and limit dose escalation.^3^ Therefore, reducing respiration‐associated motion and limiting the margin added is essential for enabling more conformal dose delivery and improving the therapeutic ratio, which leads to better treatment outcomes.^4^

Abdominal compression (AC) is a widely implemented respiratory motion‐management technique for abdominal targets, particularly when breath‐hold or respiratory‐gating strategies are unsuitable or unavailable.^5^, ^6^ Both plate‐based and belt‐based AC systems function by restricting diaphragmatic excursion, thereby inducing a shallow‐breathing state, which reduces the inferior–superior displacement of abdominal organs. By constraining the most caudal (inhalation) and cranial (exhalation) extents of motion, AC has the potential to reduce target motion amplitude and, in turn, the margins required for safe treatment planning.^7^, ^8^, ^9^, ^10^, ^11^

Numerous studies have investigated whether the compression belt (CB) consistently reduces intrafraction target respiratory motion.^12^ The reported efficacy varies considerably,^13^ often in a patient‐specific manner,^14^, ^15^, ^16^ which has motivated the adoption of adaptive approaches for patients being treated with a CB to compensate for daily anatomical and target volume changes. Online adaptive radiotherapy (oART) offers the ability to re‐optimize the treatment plan according to daily anatomical and target volume changes.^17^, ^18^ Although oART mitigates the dosimetric consequences of daily anatomical variability, it imposes substantial demands on the clinical workflow. The clinical integration of abdominal compression with MR‐guided adaptive replanning has been described for pancreatic SBRT on the MR‐Linac, where compression was used in conjunction with daily adaptation to reduce treatment volumes and manage intrafractional motion.^19^ The present study complements this workflow by characterizing the interfractional anatomical variability introduced by CB placement, a component of geometric uncertainty not addressed by intrafractional motion management alone. A clearer understanding of the magnitude and nature of interfraction anatomical variation under CB is therefore necessary to determine whether resource‐intensive daily adaptation is justified, or whether simpler margin‐based approaches remain sufficient for patients treated with compression.

Few studies have quantified interfractional volumetric or positional changes under abdominal compression. Existing work has primarily focused on plate‐based compression systems,^20^ volunteer cohorts,^21^ or single‐site evaluations.^22^ Evidence regarding belt‐based compression systems remains limited, despite mechanical differences that could produce distinct patterns of interfraction variability. Additionally, direct comparisons between CB and non‐CB patients remain limited, so therefore the degree to which belt placement affects reproducibility relative to standard setups has not been well studied. By characterizing these interfractional positional changes and integrating them with established evidence on the dosimetric consequences of organ displacement,^23^ their clinical significance can be assessed.

The purpose of this study is to evaluate the interfraction reproducibility of CB placement and the associated anatomical effects using daily treatment magnetic resonance (MR) images in a clinical patient cohort. By characterizing organ positional shifts, volumetric changes, and patient‐specific variations across fractions, and comparing these findings with those from a non‐CB cohort, this work aims to determine the extent to which CB‐related setup variability influences daily anatomy, and the need for oART to account for induced fractional changes.

## 2 METHODS

### 2.1 Patient cohort creation

The CB patient cohort was established by retrospectively collecting images from patients treated with the ZiFix Abdominal/Thoracic Motion Control System's CB between May 2024 and July 2025, under Institutional Review Board (IRB) approval. The cohort of CB patients were treated for diseases located in the liver, kidneys, abdominal nodes, or the pancreas. Inclusion criteria for the cohort required patients to have at least three daily fractions with available treatment registration files, a planning target volume (PTV) located outside of the compression region, and sufficient field of view on the daily MR treatment images to visualize both the compressed and PTV regions. For patients receiving more than six fractions, only the first six were included in the analysis to prevent overall findings from being weighted towards a single patient. Finally, patient images were required to demonstrate adequate contrast in abdominal organs to allow for consistent and accurate registration calculations.

PTVs located outside the CB region were selected for analysis, with three patients excluded whose PTVs fell within the CB region. While the institution treats patients with PTVs in both locations, the cohort with PTV's centroid outside the compression region was larger, providing greater statistical power. Importantly, this selection reflects a deliberate design choice; PTVs within the CB region would be expected to correlate more directly with CB variability, whereas PTVs outside this region allow for the assessment of whether CB motion management influences dosimetric outcomes in a less mechanistically coupled setting.

A matched cohort was created, identifying patients where AC was not employed as a motion management strategy and whose PTV was positioned within the abdominal region, thereby providing a comparable FOV for anatomical analysis relative to the CB cohort. For each candidate, age, BMI, weight, and gender were recorded. Inclusion criteria for this cohort remained consistent with those outlined above, excluding criteria specific to the use of the CB. A matched non‐CB cohort of equivalent size was then assembled through systematic patient selection, with demographic variables optimized to match the CB cohort closely. All cohort matching was completed prior to the acquisition of any measurements to eliminate the potential for confirmation bias. Additionally, neither bladder filling nor bowel preparation protocols were used for either cohort, consistent with institutional simulation practice for abdominal targets.

### 2.2 MR image acquisition

All patients in this study underwent radiation therapy treatment on the Elekta Unity (Elekta AB, Stockholm, Sweden), a system combining a 1.5T MR scanner for image guidance with a linear accelerator (MR‐Linac). Because the Unity system supports multiple clinical abdominal protocols, the pulse sequence parameters differed slightly between patients. Additional information about the sequences utilized in this study are summarized in Supplementary Material T1. Though MR scans between patients may differ, each patient received the same sequence for each fraction of their treatment, allowing for comparisons of within‐patient interfractional motion.

### 2.3 Interfraction anatomical reproducibility analysis

Daily treatment MR images, their corresponding treatment alignment registration files, and PTV structures were exported from the Monaco Treatment Planning Software (Elekta Sweden, Version 6.2.2.0) and anonymized in MIM (MIM Software, Cleveland, OH, USA). For both patient cohorts, an axial slice at the centroid of the PTV structure was selected, and an external contour was manually delineated on each daily MR. The same physicist completed all external contours, and then each contour was independently verified by a single board‐certified physicist to minimize interobserver variability. Once the contours were verified, they were subsequently transferred to the first fraction to enable quantitative comparisons in MIM. For the CB cohort, an external contour was also created using the same methodology in the region of maximum compression, indicated by the smallest visual area. Figure 5 visualizes the spatial separation of the delineated CB and PTV regions, in addition to external anatomical variations for a representative patient.

The external contour volumes were recorded and normalized by slice thickness to allow for comparison of cross‐sectional areas (CSA). These areas were de‐meaned on a per‐patient basis to center the data and assess relative changes independent of patient size.

External contours over the PTV across treatments were compared using the Dice Similarity Coefficient (DSC) and Mean Distance to Agreement (MDA) calculations in MIM. Comparisons were made between consecutive fractions as well as relative to fraction one to evaluate interfraction geometric variability and potential positional drift over the course of treatment. Both metrics were computed unidirectionally, with fraction one serving as the reference dataset, consistent with the clinical workflow in which the original treatment plan represents the baseline against which the need for adaptive replanning is assessed. While DSC is inherently symmetric and thus unaffected by this directionality, the unidirectional MDA reflects geometric deviations relative to the planning fraction, which is the clinically operative quantity of interest. These values were compared against values measured in the non‐CB cohort. External contours in the maximum compression region of the CB cohort were compared using Hausdorff Distance (HD) and DSC to characterize the reproducibility in the area directly influenced by belt placement and compression pressure.

Differences between the CB and non‐CB cohort measurements were analyzed using the Mann‐Whitney U test, with significance defined as *p* < 0.05. Additionally, the Spearman correlation test was performed to assess the relationship between values measured in the maximum compression region and the PTV region within the CB cohort.

### 2.4 Adipose tissue segmentation

**FIGURE 1:**
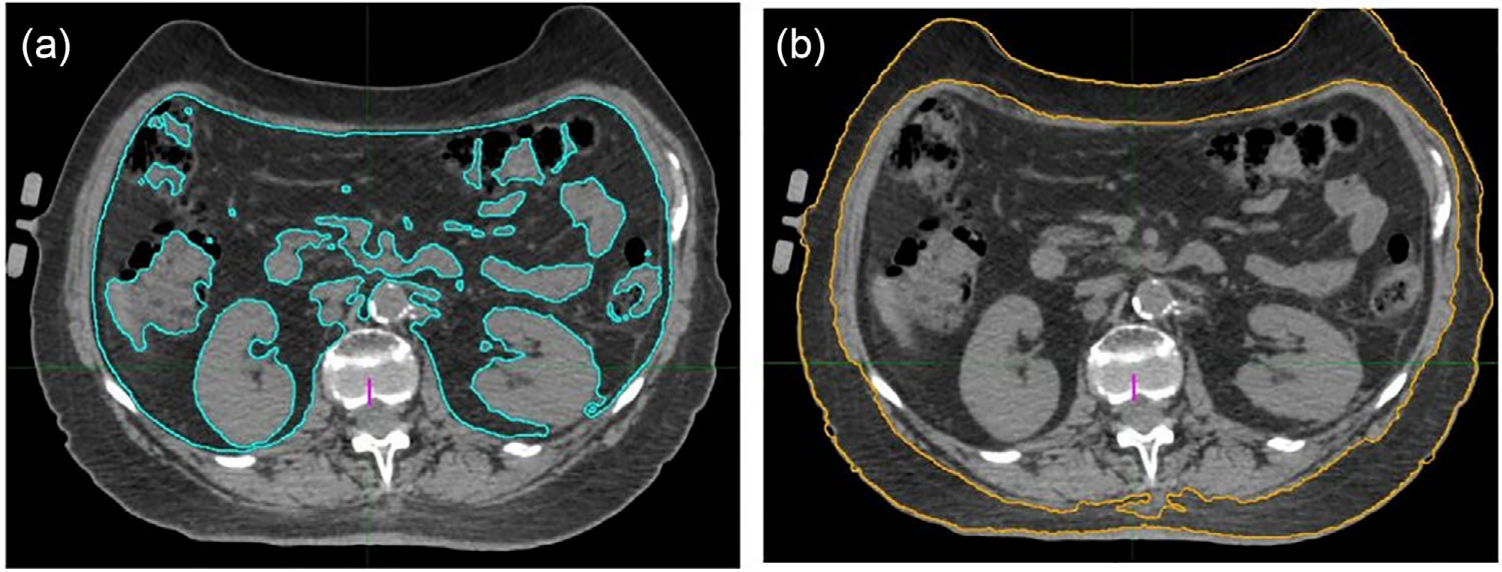
Example of adipose tissue segmentation. (a) visceral adipose tissue (VAT) and (b) subcutaneous adipose tissue (SAT).

The inclusion of subcutaneous and visceral adipose tissue volumes as covariates in the linear mixed‐effects models was informed by prior work suggesting differential effects of fat distribution on setup reproducibility in abdominal radiotherapy.^24^ To analyze the effects of fat volumes on anatomical variability, adipose tissue (AT) was delineated at two anatomical levels: the intervertebral disc between the 1st lumbar vertebra (L1) and the 2nd lumbar vertebra (L2), using methods discussed in Daley et al.,^25^ with the following changes. CB patients did not have MR images acquired without the CB; therefore, subcutaneous AT (SAT) and visceral AT (VAT) were delineated on the MR images acquired at the first treatment fraction with the CB present. External and internal anatomical boundaries were manually delineated using the same method previously described for the external contours over the CB and PTV region, and verified by a single board‐certified physicist to minimize interobserver variability. SAT and VAT regions were then generated through subtracting an intensity threshold contour from the external and internal boundary contours, followed by manual correction and visual verification. An example of delineated SAT and VAT volumes can be found in Figure 1. Volumes were automatically calculated in MIM and normalized by slice thickness.

### 2.5 Statistical modeling

To assess whether patient demographic characteristics explained interfraction variability, linear mixed‐effects models (LMEMs) were constructed for each outcome metric (de‐meaned CSA, DSC, MDA/HD) in both the PTV region and the compression region. Fixed effects included fraction number (categorical), age (z‐scored), gender, body mass index (BMI) (z‐scored), SAT volume (z‐scored), VAT volume (z‐scored). Patient ID was included as a random intercept to account for repeated observations. In models where convergence permitted, a random slope for fraction number was added to represent patient‐specific changes across treatment. Maximum likelihood estimation (ML) was used for model comparison during development. The final inferential models were fitted using restricted maximum likelihood (REML) to obtain unbiased variance estimates. Model performance was assessed using log‐likelihood, akaike information criterion (AIC), bayesian information criterion (BIC), marginal R^2^ (variance from fixed effects), and conditional R^2^ (variance from fixed + random effects). Nested model comparisons using likelihood‐ratio tests demonstrated that adding demographic covariates produced only modest improvements in model fit. For clarity of interpretation, the full demographic model was retained as the primary inferential model for all outcomes. All analyses were performed in Python (version 3.12.11) utilizing the statsmodels package for LMEM.

### 2.6 Accessing interfraction organ positioning reproducibility

Interfraction organ displacement was evaluated for three organs: liver, right kidney (Rkid), and left kidney (Lkid) on each daily treatment MR image. Each MR was originally aligned using the corresponding treatment registration file exported from the TPS. Using a rigid mutual information registration algorithm in MIM, box‐assisted alignment was performed for each organ individually, always using fraction one as the primary dataset. Deformable registration techniques were excluded to not introduce additional uncertainty caused by varying pixel intensities and contrast caused by the multiple pulse sequences utilized in this study. The registration box was confined to the organ of interest, explicitly excluding the patient surface boundary, as it would bias the registration. All registrations were visually inspected and manually adjusted as necessary. Displacement was calculated as the scalar magnitude of the three‐dimensional translation vector (in millimeters) and therefore reflects the overall extent of positional change without directionality. This process was repeated for each organ across all fractions relative to fraction 1. In the analysis, organs containing the PTV were excluded as the treatment registration is inherently optimized to align that structure, which systematically suppresses the apparent displacement of the organ.

## 3 RESULTS

### 3.1 Patient cohorts

In total, 17 patients met all established inclusion criteria. Patient demographics for both cohorts are summarized in Table 1. The CB cohort consisted of liver, pancreas, Rkid, and LKid disease sites, and the non‐CB cohort included the addition of abdominal nodes, spine, and peritoneum sites. The average slice thickness for the CB and non‐CB, taking into account the different MR pulse sequences utilized in this study, is 3.58 mm and 2.85 mm, respectively.

**TABLE 1:** Demographic comparison between CB and non‐CB patient cohorts.

	BMI	Weight (lbs)	Age (years)	Number of Patients (Men/Women)
CB	26 ± 3.4	173.76 ± 18.58	69 ± 11	17 (10/ 7)
Non‐CB	28 ± 5.2	182.29 ± 41.99	68 ± 12	17 (9 / 8)
Total	27 ± 4.4	178.03 ± 32.75	68.5 ± 11.5	34 (19 / 15)

The mean treatment duration, defined as the interval between the first and final fraction included in the analysis, was 13.8 days for the CB cohort and 9.0 days for the non‐CB cohort. The total number of fractions differed modestly between groups, 88 for the CB cohort and 77 for the non‐CB cohort, yielding mean inter‐fraction intervals of 2.76 days and 2.07 days, respectively.

### 3.2 Assessment of interfractional PTV CSA stability

**FIGURE 2:**
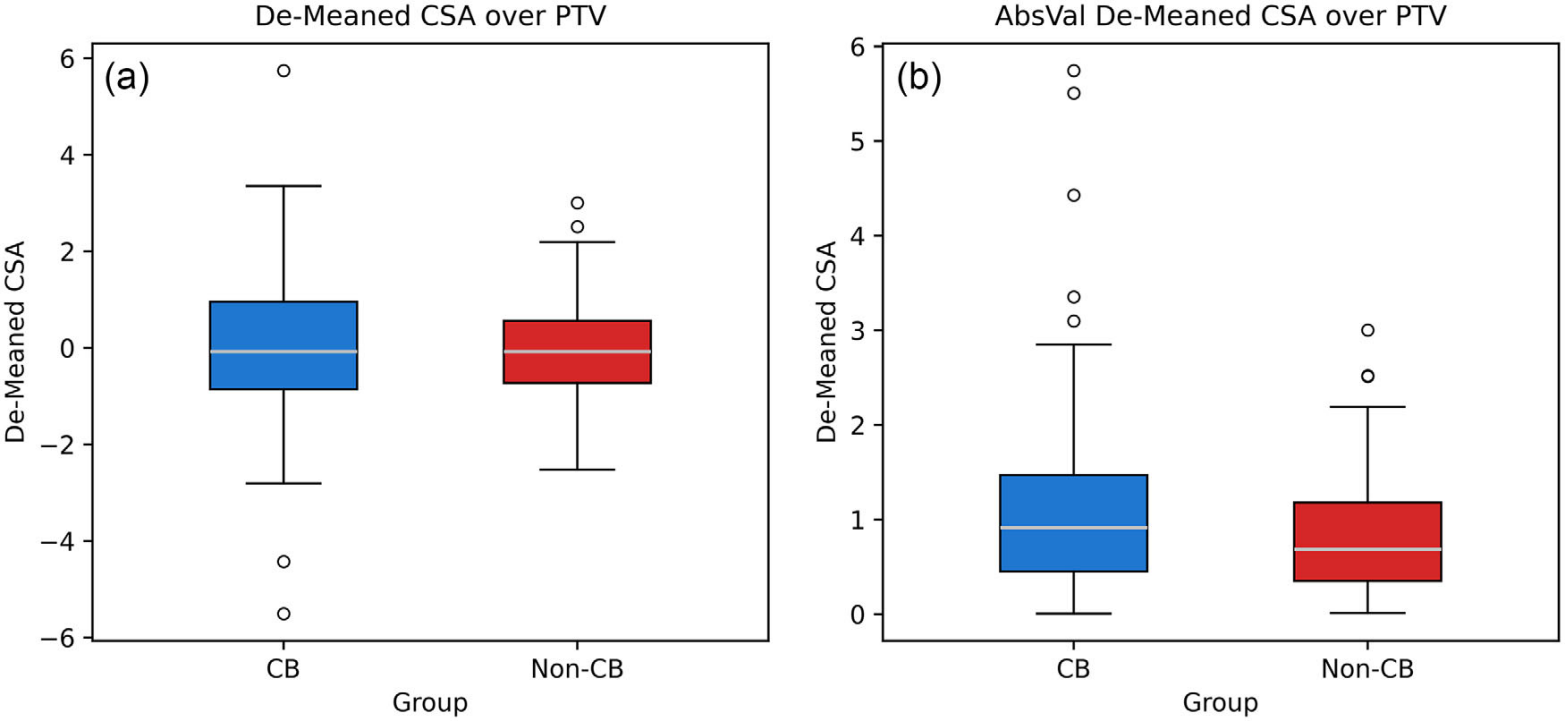
Comparison of interfractional cross‐sectional area (CSA) variability at the center of the PTV between patients treated with and without CB. (a) De‐meaned CSA values and (b) absolute value of de‐meaned CSA values. Note that the apparent change in outlier classification between panels A and B reflects the mathematical effect of the absolute value transformation on the interquartile range, and does not indicate truncation or omission of data; the full range of values is represented in both panels.

As shown in Figure 2a, no statistically significant difference was observed between the CB and non‐CB cohorts for de‐meaned CSA (cm^2^) (Mann–Whitney U test, *p* = 0.88; rank‐biserial = –0.013), indicating comparable spread of interfractional variations between groups. However, analysis of absolute deviations revealed a small but statistically significant difference (Mann–Whitney U test, *p* = 0.04; rank‐biserial = –0.178), suggesting that the CB cohort exhibits slightly greater variability in area magnitude.

**FIGURE 3:**
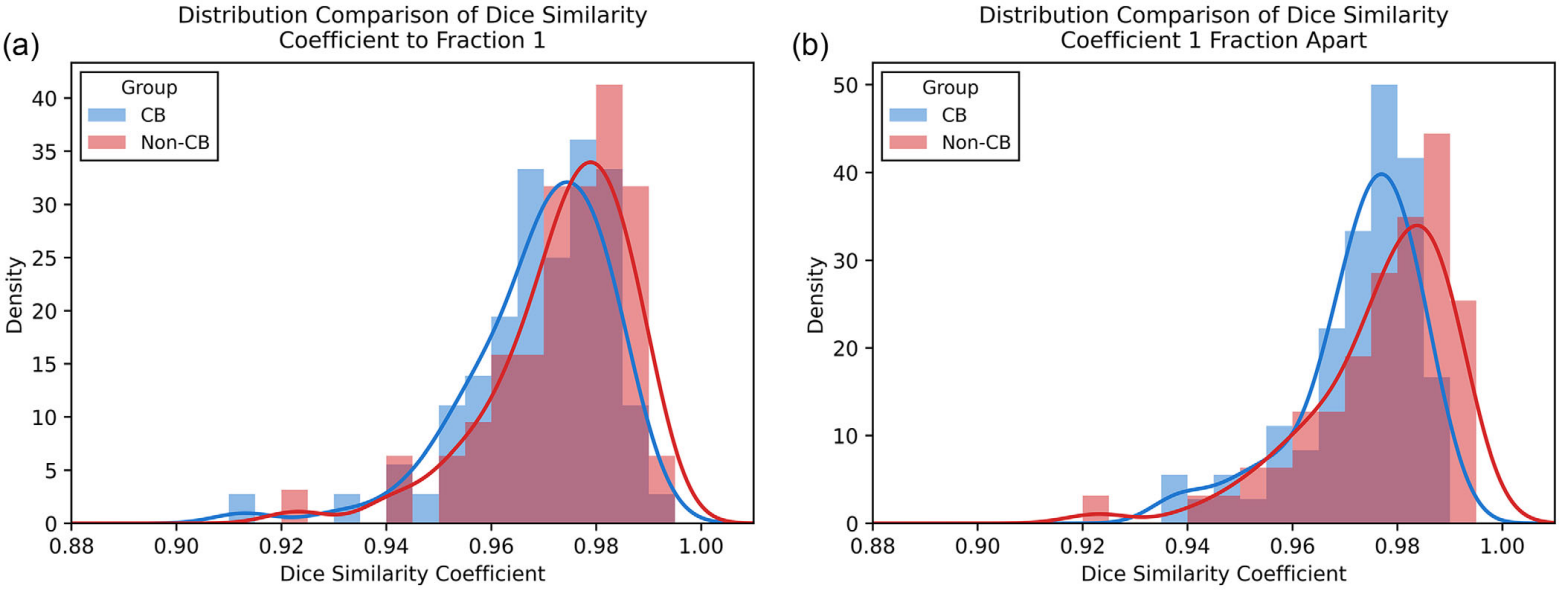
Distribution of interfraction dice similarity coefficients (DSC) for external contours over the center of the PTV in the CB and non‐CB cohorts. (a) DSC calculated between each fraction and fraction 1. (b) DSC calculated between sequential fractions. Overlaid kernel density estimate (KDE) curves provide smoothed approximations of the underlying probability density for each cohort, highlighting differences in the spread and central tendency of the DSC distributions. Statistically significant differences were observed in both distribution comparisons.

**FIGURE 4:**
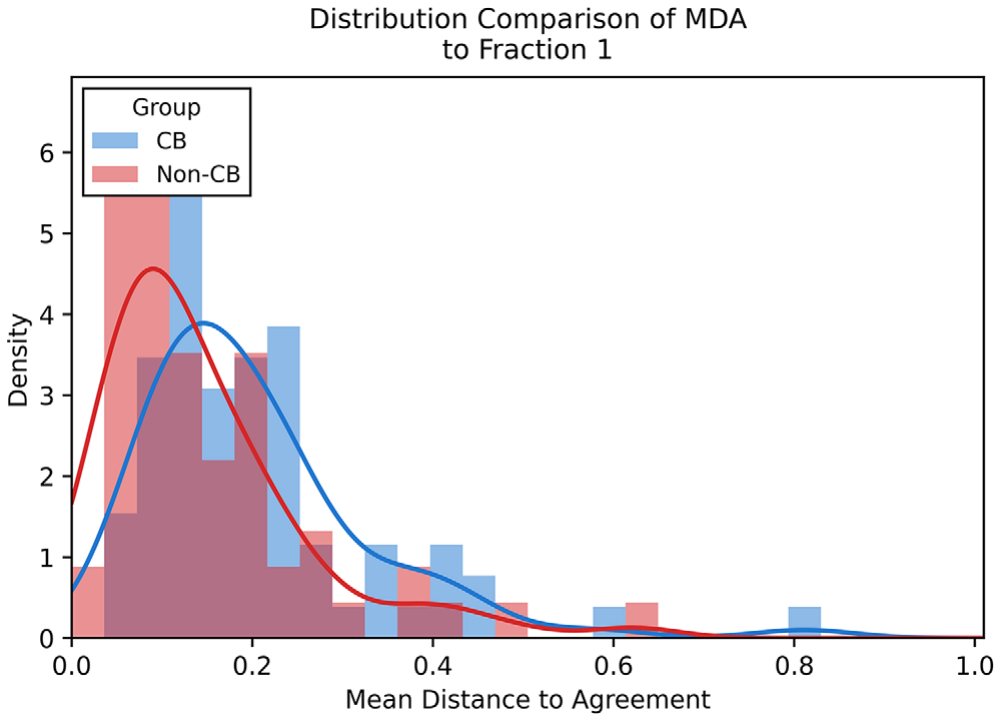
Distribution of mean distance to agreement (MDA) between each fraction and fraction one for CB and non–CB cohorts. Overlaid kernel density estimate (KDE) curves illustrate the underlying probability density for each group, highlighting differences in distribution width and tail behavior. A statistically significant difference was observed between cohorts (Mann–Whitney U, *p* < 0.001), with the CB cohort demonstrating larger interfraction variability in PTV cross‐sectional contour agreement.

Figure 3 compares the distributions of interfraction DSC between both cohorts. Statistical comparison demonstrated significant differences in DSC for Figures 3a and 3b (*p* = 0.01 and 0.03, respectively), with corresponding rank‐biserial effect sizes of 0.258 and 0.222, indicating small‐to‐moderate effects. While DSC may be less sensitive to small changes in larger volumes, such as CSA, and does not provide numerical data to interpret potential clinical significance, the MDA offers additional insight. The median MDA for the CB and non‐CB cohorts was 0.175 mm and 0.109 mm, respectively, with Figure 4 illustrating a statistically significant upward shift in the CB cohort (*p* < 0.001), reflecting greater interfractional variability in PTV CSA.

### 3.3 Maximum compression region CSA variability

**FIGURE 5:**
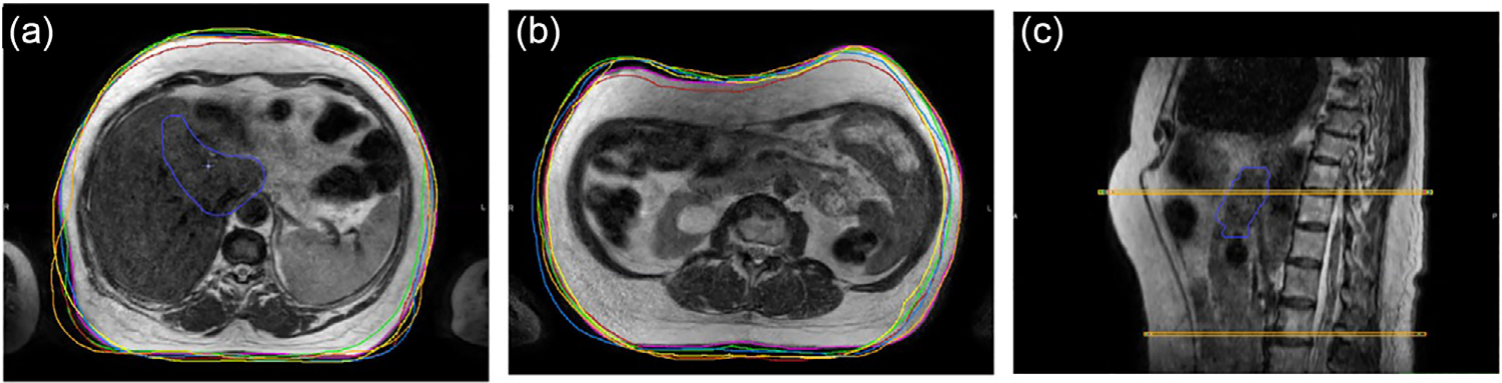
A representative patient example of external contours, drawn on each daily MR, and transferred to fraction 1. Each color represents a different fraction's external anatomy at (a) The center of the PTV (blue, PTV centroid in purple), (b) maximum compression region, (c) a sagittal view of the spatial separation.

Analysis of the maximum compression region revealed that, when comparing each fraction to fraction one, the median HD was 1.6 cm. Examination of a representative patient, illustrated in Figure 5, suggests that this displacement is directly attributable to variations in CB placement and compression shape.

The median DSC in the compression region between sequential fractions and fraction one was 0.962, indicating slightly higher variability than observed in the PTV region, which had a median DSC of 0.971.

Spearman correlation analysis demonstrated no statistically significant relationship between de‐meaned CSA values in the maximum compression region and the PTV region (*p* = 0.99), indicating that variability in the compression region does not directly translate to variability in the PTV.

### 3.4 Linear mixed‐effects modeling of interfraction variability

LMEMs were used to determine whether interfraction variability was associated with patient characteristics. Separate models were fitted for de‐meaned CSA, DSC, and MDA in the PTV region, and for de‐meaned CSA, DSC, and HD in the maximum CB region. Fraction, age, BMI, gender, SAT, and VAT were modeled as fixed effects, with patient ID as a random intercept. Nested model comparisons using likelihood‐ratio tests demonstrated that adding demographic covariates produced only modest improvements in model fit. For clarity of interpretation, the full demographic model was retained as the primary inferential model for all outcomes

Across all three PTV metrics, demographic effects were small. Fixed effects explained only a small share of the variance (R^2^_m_ = 0.06 – 0.29), whereas models that included random patient effects explained substantially more (R^2^_c _= 0.21 – 0.78). Although age, gender, and BMI reached statistical significance in some models, the corresponding effect sizes were minor, producing fractional changes in DSC and sub‐millimeter changes in MDA. Adding SAT or VAT did not improve the model fit.

De‐meaned CSA beneath the CB showed minimal dependence on demographics. The full model explained only ∼3% of variance with fixed effects (R^2^_m_ ≈ 0.03), and none of the covariates materially improved the fit. This suggests that local CSA fluctuations under the belt are patient‐specific and not systematically related to age, gender, BMI, or adiposity.

In contrast, shape‐based metrics demonstrated clearer demographic effects. For DSC in the maximum compression region, adding age, gender, and BMI improved model performance (R^2^_m_ increasing from ∼0.02 to ∼0.43), with gender and BMI contributing significantly. SAT and VAT did not provide further benefit. Although absolute coefficients remained small, these findings indicate that overlap of belt‐region contours is partially predictable.

The strongest dependencies were observed for HD. The final model explained 22% of variance with fixed effects (R^2^_m_ = 0.216) and 40% when random effects were included (R^2^_c_ = 0.403). Male gender, higher age, and higher BMI were associated with reduced HD relative to fraction one (all *p* < 0.02). SAT and VAT did not improve model fit. Quantitatively, a one‐SD increase in age or BMI corresponded to several millimeters of reduction in HD, and male patients exhibited ≈5 mm lower HD on average.

Although demographic factors modestly affected reproducibility of the CB contact shape, they did not translate into improved stability at the PTV. Compression‐region and PTV‐region metrics were not strongly correlated, and PTV variability remained dominated by random patient‐level effects. A summary of these results are outlined in Table 2.

**TABLE 2:** Summary of linear mixed‐effects model (LMEM) results for PTV and compression belt (CB) regions.

Region	Metric	Demographic Effects (significant only)	Direction of Effect	Variance Explained (R^2^_m_ / R^2^_c_)
PTV	De‐Meaned CSA	Age (*p* < 0.01)	Older = smaller metric	0.06 / 0.21
PTV	Dice to F1	Age, Gender, BMI (all *p* < 0.05)	Older, Male, Higher BMI = slightly higher metric	0.29 / 0.78
PTV	MDA	Age, Gender, BMI (all *p* < 0.05)	Older, Male, Higher BMI = slightly smaller metric	0.24 / 0.75
CB	De‐Meaned CSA	None	No directional pattern	0.03 / 0.15
CB	Dice to F1	Gender (*p* = 0.01), BMI (*p* < 0.01)	Male, Higher BMI = higher metric	0.43 / 0.61
CB	HD	Age, BMI (both *p* < 0.01), Gender (*p* < 0.05)	Older, Male, Higher BMI = smaller metric	0.22 / 0.40

### 3.5 Interfractional organ positional changes

**FIGURE 6:**
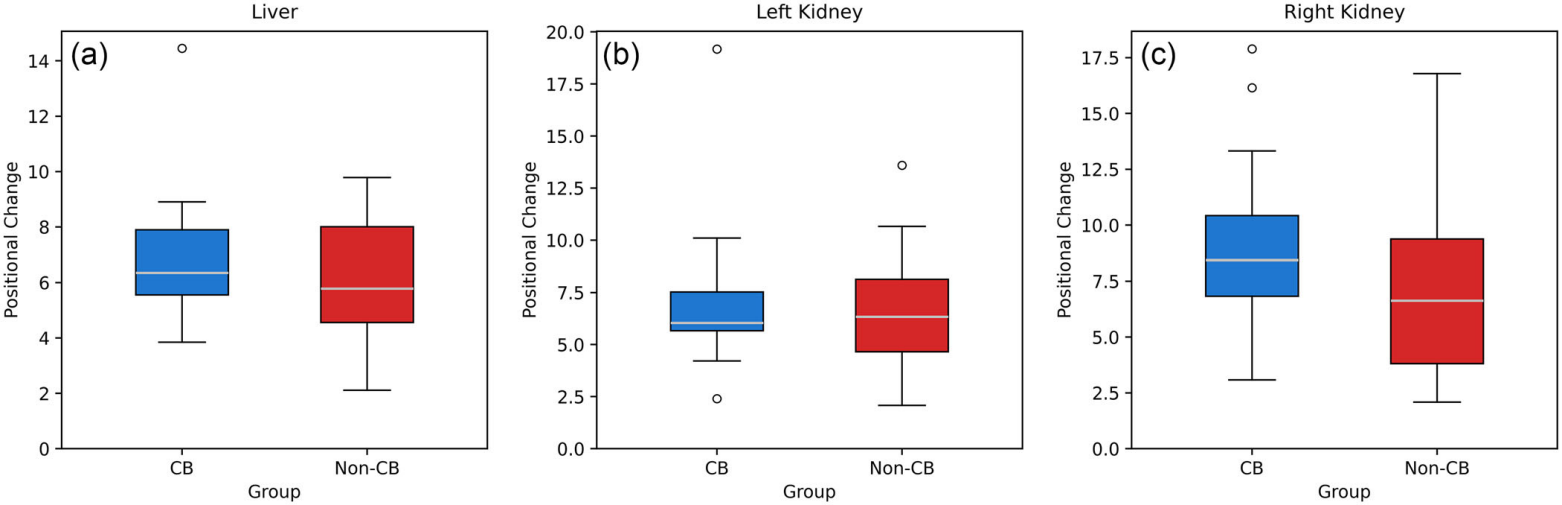
Interfractional organ positional displacement for a) liver b) left kidney c) right kidney in CB and non‐CB cohorts. Box‐and‐whisker plots show the distribution of Euclidean displacements relative to fraction one for each organ, disregarding organs where the PTV is present. As a scalar quantity, Euclidean displacement does not convey directional information. No statistically significant differences were observed between cohorts (Mann‐Whitney U, all p > 0.05).

Interfractional displacements of the liver, Rkid, and Lkid were compared between CB and non‐CB cohorts (Figure 6). No statistically significant differences between cohorts, with the Rkid showing the closest trend toward significance (*p* = 0.11).

Table 3 summarizes the mean fractional positional differences for each organ, ranging from 5.91 mm for the non‐CB liver to 9.15 mm for the CB Rkid. These findings suggest that, while interfractional organ motion exists, the use of the CB does not significantly alter overall organ displacement in this cohort.

**TABLE 3:** Average interfractional positional changes of the liver and kidneys for CB and non‐CB cohorts.

Organ	Average Positional Changes (mm)	
	CB	Non‐CB
Liver	7.15 ± 2.83	5.91 ± 2.18
Left Kidney	7.08 ± 3.51	6.65 ± 2.85
Right Kidney	9.15 ± 3.92	6.86 ± 3.74

## 4 DISCUSSION

The present study evaluated the reproducibility of patient setup and organ positioning with and without the use of a CB during abdominal MR‐guided radiotherapy. Registration files demonstrated the optimal achievable treatment alignment for abdominal target sites, given the soft tissue contrast achievable using MR images. The comparison between a CB cohort and a non‐CB cohort provided the ability to draw conclusions of fractional variations introduced by the CB compared to baseline, expected changes to gauge not only statistical significance, but also clinical implications.

Interfraction DSC scores over the PTV region were significantly lower in the CB cohort compared with the non‐CB cohort. Although the absolute differences were small, reduced DSC and elevated MDA in the CB cohort indicate subtle but measurable day‐to‐day changes in external contour geometry. The dosimetric consequences of these geometric differences were not evaluated in the present study; whether they translate to clinically meaningful changes in target coverage or an organ‐at‐risk (OAR) dose requires direct dosimetric analysis and should be the subject of future work.

Interfractional shape variability in the maximum compression region was greater than that observed at the PTV level; however, no statistically significant correlation was found between the two regions. For targets located outside the compression zone, belt‐induced local deformation in the compression region did not propagate to the PTV in a predictable or correlated manner. This finding should not be interpreted as a generalizable anatomical principle; rather, it reflects the specific study design in which PTVs were required to be spatially separated from the compression region. The Spearman correlation between compression‐region and PTV‐region CSA variability should be interpreted with caution given the sample size of 17 CB patients, which may be insufficient to detect a true relationship of modest effect size.Analysis of interfractional CSA changes at the center of the PTV indicated no significant differences in variability between CB and non‐CB cohorts. While variations in cross‐sectional area have the potential to influence dosimetric parameters such as integral dose, this relationship was not explicitly valuated. The observed de‐meaned CSA values and absolute deviations were small in magnitude, and well within tolerances typically managed through daily image guidance and oART.

It should be acknowledged that the external contour analysis was performed on a single axial slice per anatomical region. Although this methodology facilitated consistent and reproducible comparisons across a heterogeneous patient cohort with variable imaging parameters, it does not capture volumetric changes along the superior‐inferior extent of the target or CB region, representing an inherent limitation in this approach.

The sub‐millimeter MDA observed at the PTV level in the present study is not directly comparable to the centimeter‐scale internal organ deformations reported by Eccles et al. using plate‐based compression and deformable registration on liver structures.^20^ The present analysis quantifies external contour variability at a PTV located outside the compression zone, a fundamentally different quantity from internal organ deformation under direct compressive load. Consistent with prior literature, the HD of 1.6 cm observed in the maximum compression region of the present cohort reflects deformation magnitudes comparable to those reported by Eccles et al. and Lee et al.; the critical finding of the present study is that this compression‐zone variability does not propagate proportionally to spatially separated targets.^20^, ^21^ Mechanical differences between plate‐based and belt‐based AC systems may further contribute to differences in deformation magnitude and spatial distribution.

The null findings reported by the Manchester group in 8 participants are likely attributable to insufficient statistical power to detect the small‐to‐moderate effect sizes observed in the present study.^26^ Furthermore, volume‐based metrics evaluated in that study may be less sensitive to subtle contour‐level shape variability than the DSC and MDA measures employed here, as organ volume can remain stable while contour geometry changes measurably under repeated compression.

Older patients, patients with higher BMI, and male patients demonstrated more stable belt‐contact shape across fractions in the region of maximum compression. The modest explanatory power of demographic variables, including BMI, in predicting interfractional variability in the compression region is consistent with findings by Daly et al., who similarly reported no significant correlation between BMI or abdominal adipose tissue and compression effectiveness in abdominal radiotherapy.^25^ In the CB region, higher BMI and male gender were associated with reduced HD and improved DSC, suggesting that patients with greater body mass may achieve more consistent belt contact geometry, potentially due to a larger soft tissue buffer attenuating day‐to‐day variation in belt positioning. These findings partially align with Price et al., who identified adipose tissue distribution as a potential contributor to daily IGRT setup variability in abdominal radiotherapy patients.^24^ However, whereas Price et al. highlighted the differential role of visceral versus subcutaneous fat compartments, the addition of SAT and VAT as covariates in the present models did not improve fit beyond BMI alone, suggesting that gross body habitus rather than fat distribution pattern is the primary body composition driver of compression‐region reproducibility.

However, this predictability did not translate to the PTV region, indicating that anatomical changes due to local belt positioning reproducibility are not directly correlated with changes in the PTV region, when the target is spatially separated from the compression region. This finding likely reflects a study design limitation imposed by the patient cohort inclusion criteria, which required PTVs to be located outside the compression region. Consequently, demographic information alone is insufficient to guide patient selection for compression or to reduce image‐guidance requirements. Interfraction variability should continue to be managed through MR‐based setup alignment and, when indicated, adaptive planning to maintain dosimetric robustness in the presence of millimeter‐scale anatomical changes.

Similarly, demographic characteristics did not meaningfully predict PTV CSA reproducibility. Although some effects reached statistical significance, their magnitudes were small and explained only a fraction of the total variance. This reinforces that interfraction variability is dominated by patient‐specific factors likely related to anatomy, breathing mechanics, or day‐to‐day setup rather than age, BMI, adiposity, or gender.

Interfraction organ displacement analysis further supports these conclusions. No statistically significant differences in liver or kidney motion were observed between cohorts, although the Rkid demonstrated the closest trend toward significance (*p* = 0.11). The absence of statistical significance does not preclude clinical relevance. Although the dosimetric effects of the observed positional changes were not directly evaluated due to the heterogeneity of the PTV location in the CB cohort, small interfractional displacements remain clinically relevant when an organ lies near a steep‐dose gradient, particularly along the organ boundary that is the primary determinant of dosimetric impact. The use of rigid registration to quantify organ positional changes represents a methodological limitation, as organ deformation can not be characterized, potentially limiting the quantification of clinically meaningful changes that would otherwise inform the need for adaptive intervention. Furthermore, because rigid registration inherently averages motion across the structure, millimeter‐level differences may underestimate surface proximity at dose‐limiting interfaces. Finally, the small difference in mean slice thickness between the two cohorts introduces differential sensitivity along the superior‐inferior axis during registration. These observations reinforce the importance of image‐guided alignment and, when appropriate, adaptive replanning to ensure healthy tissue sparing. Standard geometric margins are designed to accommodate small interfraction motion, but may require careful verification for abdominal targets where millimeter‐level displacements can be dosimetrically meaningful.

A mechanistically important observation emerges from the comparison of the contour‐based and organ displacement results: DSC and MDA were significantly worse in the CB cohort, while rigid organ displacement showed no statistically significant difference between groups. Chu et al. similarly reported no significant difference in setup displacement between CB and non‐CB patients, a finding that may in part reflect the systematic reshaping of the abdominal contour induced by CB placement.^27^ The CB physically deforms the abdomen into a concave profile, creating a geometrically altered surface that rigid registration may partially accommodate through translational and rotational corrections, effectively masking the underlying shape change within displacement metrics. Contour‐based metrics such as DSC and MDA are not subject to this compensation and are therefore more sensitive to the day‐to‐day reproducibility of this deformed geometry. Collectively, these results suggest that CB use preferentially introduces non‐rigid shape variability rather than bulk translational displacement, and that rigid registration alone may systematically underestimate the true extent of CB‐induced anatomical change. This has important implications for image‐guidance strategies in CB patients, further supporting the role of MR‐based soft tissue assessment and adaptive replanning to detect and respond to variability that rigid displacement metrics would not capture.

The Unity MR‐Linac's adaptive workflow addresses this interfractional variability by allowing daily plan re‐optimization to the updated anatomy, which is precisely why this platform is particularly well‐suited for CB patients. It should be noted that there is no universally established dosimetric threshold for triggering adaptation currently exists for abdominal MR‐Linac treatments; adaptation decisions are made through per‐fraction visual and dosimetric assessment of the daily MR image relative to the reference plan. The observed interfractional differences are clinically meaningful when considered in the context of non‐adaptive platforms: on a conventional cone beam computed tomographyguided system, soft tissue contrast is insufficient to detect the subtle anatomical changes characterized in this study, and adaptive replanning is unavailable. Furthermore, the non‐CB cohort provides a clinically relevant reference for the degree of anatomical variability considered acceptable under standard, non‐CB setups, against which the additional burden introduced by the CB can be contextualized. This study therefore supports the argument that CB patients specifically benefit from MR‐guided adaptive radiotherapy, not because adaptation is routinely triggered, but because the platform provides the sensitivity and capability to detect and respond to CB‐induced anatomical changes when they occur.

### 4.1 Limitations

Several limitations of this study warrant acknowledgment. The cohort was relatively small (*n* = 17 per group), which may limit statistical power, particularly for subgroup analyses and the detection of modest effect sizes such as the Spearman correlation between compression‐region and PTV‐region variability. The mixed disease‐site composition of both cohorts introduces additional heterogeneity, as interfractional anatomical variability may differ across tumor locations and treatment volumes, limiting the generalizability of findings to specific anatomical sites. The difference in mean treatment duration between cohorts (13.8 days for CB versus 9.0 days for non‐CB) was not a controlled matching variable and may have introduced temporal confounds into the interfractional analysis, despite the applied cap on maximum fractions included per patient.

Fractionation schedules were also not controlled as a matching variable between cohorts, reflecting the heterogeneous disease site composition. Although fraction number was included as a categorical fixed effect in all LMEMs to account for within‐patient temporal trends, between‐patient differences in fractionation intensity and overall treatment course length were not explicitly modeled. The influence of fractionation schedule on interfractional anatomical variability, therefore cannot be fully disentangled from the CB‐related effects reported. Serial patient weight measurements throughout the treatment course were not reliably documented in the institutional record for either cohort, precluding direct evaluation of weight loss as an independent contributor to interfractional anatomical variability. Given the multi‐week treatment durations observed in this study, progressive weight change remains a plausible confounding factor, particularly for CSA and external contour metrics.

Concurrent systemic therapy status was not used as a matching variable or included as a covariate in the LMEMs. While systemic therapy does not directly manifest on MR imaging, it may indirectly influence patient‐level factors such as bowel gas patterns or positioning comfort, representing an additional unmodeled source of patient‐level variability.

The external contour analysis was performed on a single axial slice per anatomical region, which, while enabling consistent and reproducible comparisons across a heterogeneous cohort with variable imaging parameters, does not capture volumetric changes along the superior‐inferior extent of the target or compression region. Manual delineation of external contours, despite verification by a board‐certified physicist, introduces a source of interobserver variability that cannot be fully eliminated, particularly at anatomical boundaries where tissue contrast is reduced.

Organ displacement was quantified using rigid registration, which captures translational and rotational components but cannot characterize organ deformation, local shape change, or non‐rigid motion. This represents a methodological limitation, as CB use may preferentially introduce shape‐level variability, consistent with the DSC and MDA findings, that rigid registration systematically underestimates. Deformable registration was deliberately excluded due to the heterogeneous pulse sequences utilized across the cohort, which introduce variable voxel intensities and organ boundary contrast that would reduce the reproducibility and reliability of deformable registration results.

This study did not evaluate intrafractional respiratory motion, which represents one of the primary clinical motivations for CB use. The efficacy of the CB in reducing intrafractional motion has been characterized in prior literature,^7^, ^8^, ^9^, ^10^, ^11^, ^12^, ^13^, ^14^ and the present work is intended as a complementary characterization of the distinct and undercharacterized interfractional component of CB‐related geometric uncertainty. Finally, the absence of direct dosimetric evaluation, adaptive replanning outcome data, or toxicity analysis limits the ability to quantify the clinical impact of the observed geometric variability. This analysis was unfeasible due to the heterogeneity of the cohort's treatment site, fractionation, prescription, and OAR limitations. Future work incorporating deformable registration, volumetric contour analysis, dose accumulation, and prospective adaptive replanning decision data would provide a more comprehensive assessment of the dosimetric consequences of CB‐related interfractional geometric variability.

## 5 CONCLUSION

The CB is employed primarily to limit intrafractional respiratory motion amplitude, reducing the margin required to account for target displacement during beam delivery. However, as this study demonstrates, CB use introduces interfractional anatomical variability, in external contour shape, cross‐sectional area reproducibility, and geometric agreement compared to a baseline non‐CB cohort. Significant differences were measured over the PTV region, where even small positional and volumetric variations have the potential to be dosimetrically relevant, though direct dosimetric evaluation is required to confirm the magnitude of this impact and is a recommended direction for future work. It was determined that, while demographics can partially explain some of the interfractional variability, particularly over the maximum CB region, it is an insufficient guide to predicting the variability caused by CB placement. Interfraction organ displacement was not statistically significant between compressed and non‐compressed patients, likely attributable to the small cohort size, but clinical significance is not negligible as accurate and safe treatment delivery depends on millimeter accuracy. Lastly, with the absence of dosimetric findings to determine clinical significance, interfractional geometric variability should continue to be managed through MR‐based setup alignment and, when indicated, adaptive planning to maintain dosimetric robustness in the presence of anatomical variability caused by the CB.

## AUTHOR CONTRIBUTIONS

All authors contributed to the manuscript preparation, final editing, and review. Data acquisition, analysis: Morgan Aire, Christopher Schneider, David Solis. Data Interpretation: Christopher Schneider, Krystal Kirby. Supervision: Christopher Schneider, David Solis, Krystal Kirby. Methodology Development: Morgan Aire, David Solis, Krystal Kirby, Christopher Schneider

## CONFLICT OF INTEREST STATEMENT

The authors have no relevant conflicts of interest to disclose.

## ETHICS STATEMENT

Retrospective analysis of patient imaging data was approved by the institutional review board (IRB) under protocol IRBAM‐25‐0203.

## Supporting information



Supporting Information

## Data Availability

The data supporting the findings of this study are available upon reasonable request from the corresponding author.
