# Supplementary material for: Evaluation of inter‐fractional anatomical variations due to compression belt placement reproducibility using MR‐guided radiotherapy treatment fraction images
Source: J Appl Clin Med Phys. 2026 Sep 3;27(9):e70783. doi: 10.1002/acm2.70783 (PMC13540267; doi:10.1002/acm2.70783)
Supplement: Supplementary file 1 [file ACM2-27-e70783-s001.pdf]

Protocol Name	T1 3D VaneXD	T2 3D Tra	T1 3D BH	T1 3D Tra	B3DVaneXD	T1 3D VaneXD SPAIR
**Number of Patients**	10	11	6	4	1	2
**Technique**	3D VaneXD	TSE	FFE	FFE	3D VaneXD	3D VaneXD
**Slice Orientation**	Transverse	Transverse	Transverse	Transverse	Transverse	Transverse
**In-plane resolution (mm)**	[0.781, 0.781]	[0.776, 0.776]	[0.862, 0.862]	[0.783, 0.783]	[0.781, 0.781]	[0.781, 0.781]
**Reconstruction Matrix**	[640, 640]	[576,576]	[560, 560]	[576, 576]	[640, 640]	[640, 640]
**Slice Thickness (mm)**	3	2.4	5	3	3	3
**Flip angle (degrees)**	15	90	10	15	40	20
**Repetition Time (TR)**	3.9 ms	2100 ms	6.2 ms	7.6 ms	3.3 ms	3.9 ms
**Echo Time (TE)**	1.2 ms	212 ms	4.0 ms	4.5 ms	1.3 ms	1.2 ms
**Scan Duration**	6 min 1 sec	3 min 20 sec	18 sec	3 min 42 sec	4 min 7 sec	4 min 31 sec
